# Combination of amyloid and FDG PET for the prediction of short-term conversion from MCI to Alzheimer´s disease in the clinical practice

**DOI:** 10.1007/s00259-025-07275-2

**Published:** 2025-04-21

**Authors:** Beatriz Echeveste, Elena Prieto, Edgar Fernando Guillén, Adolfo Jimenez, Genoveva Montoya, Rafael Villino, Mario Riverol, Javier Arbizu

**Affiliations:** 1 Clínica Universidad de Navarra, calle pio XII 36. Pamplona, Madrid, España; 2 Clínica Universidad de Navarra, Madrid, España

**Keywords:** Amnestic mild cognitive impairment (aMCI), Alzheimer’s disease (AD), Short-term conversion, Amyloid-PET, FDG-PET

## Abstract

**Purpose:**

Amnestic mild cognitive impairment (aMCI) is considered a precursor to Alzheimer’s disease (AD). Since cerebral amyloid aggregation and neurodegeneration can be detected at an early stage, it can serve as a diagnostic aid. This study aimed to determine the predictive value of Amyloid-PET and FDG-PET in determining progression to AD among patients with aMCI.

**Methods:**

This study recruited 145 patients with aMCI from October 2013 to March 2021. The patients were classified into four groups based on whether Amyloid-PET (A) and FDG-PET (N) were positive (+) or negative (-). The patients were then clinically followed to establish progression to dementia due to AD.

**Results:**

Amyloid-PET demonstrated high sensitivity (100% in year 1, 94.67% in year 4) and a high negative predictive value (100% in year 1, 88.24% in year 4). FDG-PET exhibited a high negative predictive value initially (94.59% in year 1), and during follow-up, both specificity (85%) and positive predictive value (88%) increased. The conversion from aMCI to AD had a global mean time of 39.95 months. However, progression to AD was slower in amyloid-negative patients versus amyloid-positive patients (75.07 [CI 56.54–81] vs. 32.59 months [CI 20.56–40.74] months). Taking both tests together, the time to conversion was faster in A+/N + versus A+/N- patients (27.79 [CI 20.40–33.21] vs. 37.38 [CI 20.73–48.26] months).

**Conclusions:**

Among patients with aMCI, those with a positive Amyloid-PET and an AD pattern on FDG-PET progressed to dementia significantly earlier versus those with a positive Amyloid-PET only. Using both biomarkers during the initial diagnosis enhances the prediction of short-term conversion.

**Clinical trial number:**

Not applicable. It is not a clinical trial.

## Introduction

Amnesic mild cognitive impairment (aMCI) is considered a predementia stage of Alzheimer’s Disease (AD), but not all subjects with an aMCI have prodromal AD [1]. Thus, establishing an early diagnosis and predicting conversion to AD are important in the context of effective medical care and treatment planning. Positron emission tomography (PET) has emerged as an essential tool for identifying and monitoring pathological changes within the brain. In fact, the predictive power of Amyloid-PET and 18 F-Fluorodeoxiglucose PET (FDG-PET) for AD has been established among patients with aMCI. Multiple studies have demonstrated that the presence of amyloid deposits on PET is strongly associated with a higher risk of progression from aMCI to AD dementia [1]. A meta-analysis by Jansen et al. in 2020 concluded that Amyloid-PET has high specificity (close to 90%) for predicting the conversion from aMCI to AD among amyloid-positive individuals [2]. Moreover, a longitudinal study by Vlassenko et al. found that patients with aMCI who tested positive on Amyloid-PET had a significantly higher risk of progression to AD versus amyloid-negative individuals [3]. However, FDG-PET measures cerebral metabolism and can identify patterns of hypometabolism associated with AD. Several studies have shown that the pattern of cerebral hypometabolism detected on FDG-PET can identify patients with aMCI who progress to AD and those who remain stable [4]. In line with this, a longitudinal study by Prestia et al. identified a specific pattern of hypometabolism in brain regions characteristically affected in AD that could predict the conversion from aMCI to AD with high sensitivity and specificity [5]. Therefore, both Amyloid-PET and FDG-PET provide complementary and valuable information for predicting the risk of progression from aMCI to AD.

Experts have proposed an algorithm for the differential diagnosis of cognitive impairment based on both Amyloid-PET and FDG-PET. Amyloid-PET is initially recommended for determining the short-term prognosis of aMCI. If positive, FDG-PET is added because the combination of both biomarkers is expected to predict progression more accurately and earlier versus one biomarker alone [6]. In addition, a European multidisciplinary taskforce created the first patient-centred diagnostic workflow that aims to prioritise testing for available biomarkers among patients at memory clinics. Cerebrospinal fluid biomarkers were recommended as the first-line diagnostic test for cases of AD dementia, while both Amyloid-PET and FDG-PET can be performed as second-line tests [7].

Although the individual predictive roles of amyloid-PET and FDG‐PET in aMCI are well-documented [3–4], the optimal integration of both these biomarkers in clinical practice remains unclear. Previous studies have primarily focused on the individual utility of each imaging modality, but emerging evidence suggests their improved short‐term prognostic accuracy when used in combination [6]. Thus, we aim to evaluate not only the individual contributions of amyloid‐PET and FDG‐PET but also their additive value when used in combination. This can offer deeper insights into the timing and application of these neuroimaging tools, which can guide clinicians in selecting the most appropriate diagnostic strategy for predicting conversion to AD. The short-term prognosis of aMCI is critical, because emerging disease-modifying treatments can potentially alter the disease course [8–11]. By identifying patients who are likely to progress earlier, treatment can be initiated before dementia becomes fully established. This longitudinal study aimed to determine the individual and combined accuracy of Amyloid-PET and FDG-PET in predicting the conversion to AD dementia among patients with aMCI in a real-world clinical setting. Additionally, we aimed to evaluate the need for using both biomarkers to accurately predict short-term conversion to AD.

## Materials and methods

### Study design and participants

This study retrospectively evaluated patients with a clinical diagnosis of aMCI at the Memory Unit of Clínica Universidad de Navarra from October 2013 to March 2021. All participants were assessed by a neurologist experienced in cognitive disorders. The initial assessment included a medical history review, an interview with a family member or friend, a neurological examination, laboratory tests (i.e. complete blood count, biochemical tests, vitamin B12, serum folate, glucose, lipids, syphilis serology and thyroid function), brain magnetic resonance imaging (MRI) and a neuropsychological assessment. The exclusion criteria were as follows: age > 85 years, evidence of depression or dysthymia, abnormal laboratory tests, significant medical illnesses, substance abuse that could interfere with cognitive functioning, other causes of focal or diffuse brain damage (e.g. lacunae and extensive cerebrovascular disorders evident on MRI), and follow-up of less than 6 months. The final analysis included 145 patients with a clinical diagnosis of aMCI based on the 2011 National Institute on Ageing and Alzheimer’s Association (NIA-AA) diagnostic guidelines [12]. The diagnostic criteria included a subjective memory complaint corroborated by an informant, objective evidence of memory impairment on standardised neuropsychological assessments, preserved overall cognitive function, and largely intact activities of daily living. After the clinical diagnosis, all patients underwent amyloid deposition PET scanning as part of the routine diagnostic process, and 133 patients additionally had an FDG-PET scan. Finally, all patients were followed up until January 2022 to evaluate conversion to AD.

As part of the standard practice in our routine clinical care, all participants provided written informed consent at the time of the FDG-PET scan, explicitly agreeing that their data could be used for future research studies. This study was evaluated by the Ethics Research Committee of the University of Navarra and and no ethical concerns were raised for publication.“.

### Neuropsychological assessment

All study subjects were evaluated by a trained neuropsychologist. Global cognitive function was assessed using the Mini-Mental State Examination (MMSE) [13]. Functional independence was rated using the Interview for Deterioration in Daily Living in Dementia scale (IDDD) [14]. Depression was assessed using the Geriatric Depression Scale (GDS) [15]. Several neuropsychological tests were used to assess various cognitive domains, including memory, language, attention, executive function, psychomotor speed and constructional praxis. Verbal and visual episodic memory were examined using the Free and Cued Selective Reminding Test [16] and the delayed recall of two simple figures (Massachusetts General Hospital, Boston). Attention, processing speed and executive function were assessed using Raven’s Progressive Matrices [17], the verbal fluency task (words starting with the letter ‘P’), the Stroop test [18], the Digit Span subtest of the WAIS-III [19] and the Trail Making Test parts A and B [20]. Language was evaluated using the Boston Naming Test (BNT) [21] and semantic fluency (naming animals). Constructional praxis was tested by having the subjects copy two simple figures.

### PET imaging

#### Amyloid-PET imaging

##### Patient preparation, protocol for Radiotracer administration, and acquisition and processing of images

No specific preparation of the patient or drug withdrawal was required for the amyloid PET scan. Peripheral venous access was established in the upper extremity using a catheter, and the recommended dose of the amyloid radiotracer was administered according to its technical specifications. PET was performed according to the technical data sheet of each radiotracer and following the procedure guidelines of Amyloid-PET imaging [22], using one of three radiotracers commercially available in Spain. Florbetapir was employed 79 times, Florbetaben 20 times, and Flutametamol 46 times throughout the study The PET scan was performed 50 min after 370 MBq injection of [18 F]-Florbetapir, 90 min after 240–360 MBq of [18 F]-Florbetaben, and 90 min after 185 MBq of [18 F]-Flutemetamol.

The image acquisition method was 3D on the Biograph mCT. Images were reconstructed using iterative algorithm with time of flight (TOF) and point spread function (PSF), 3 iterations, 21 subsets, a 2 mm Gaussian filter and a zoom factor of 2.

The images were subject to three methods of analysis: classic visual analysis, visual analysis assisted by a normative database, and quantitative analysis using the standardised uptake value ratio (SUVr). For the quantitative analysis, a positive test was determined using the following cut-off values: Florbetapir: > 1.17, Florbetaben: > 1.35 and Flutemetamol: > 0.6 Patients were classified based on classic visual analysis (the most commonly used method in clinical practice) as either negative (A-) or positive (A+) for amyloid deposition, which suggest a low and high probability of AD, respectively. Visual analysis was conducted followed the manufacturer’s guidelines for each radiotracer, using the syngo.via^®^ (Siemens) platform, by two independent nuclear medicine specialists blinded to clinical data (Kappa 0.936 [CI 95%: 0.862–0.985])). The contrast between the high activity in the white matter and the tracer uptake in the gray matter was systematically evaluated. In negative amyloid PET scans, nonspecific tracer retention was observed in the white matter, creating a pattern of multiple concave, arborized ramifications that did not extend into the cortical ribbon. In contrast, positive amyloid PET scans demonstrated tracer uptake in the gray matter, resulting in a blurring of the gray-white matter junction due to tracer retention extending into the neocortex. This uptake formed a smooth and well-defined boundary at the outermost edge of the cerebral cortex [23–25]. In cases of diagnostic discrepancies, a consensus diagnosis was reached.

#### FDG-PET imaging

FDG-PET imaging was performed according to the EANM (European Association of Nuclear Medicine) guidelines [26]. Image acquisition was performed using the same PET/CT scanner as in the amyloid study. Images were reconstructed using an iterative algorithm with time of flight (TOF), 3 iterations, 21 subsets, a 3-mm Gaussian filter and a zoom factor of 2. The images were independently analysed by two Nuclear Medicine specialists (Kappa 0.624 [CI 0.492-0.751]) trained in brain PET images. The evaluators were blinded to the clinical and pathologic diagnoses, and each study was compared using a normality database as a reference to aid in visual analysis. The DatabaseComparison software (Siemens^®^) was used. This software calculates cerebral metabolism using a voxel-wise morphometric approach and compares it with a database of normal studies. Statistically significant differences are represented through a 3D stereotactic surface projection (3D-SSP) displayed in two groups of eight images.

Image 1: example of positive amyloid PET. Flobertaben SUVr analysis mean 1.90.

The results were categorised into two diagnostic groups: N + and N−. Patterns compatible with AD (N+) were characterised by bilateral temporal or parietal hypometabolism or both, highly asymmetric temporoparietal hypometabolism, or posterior cingulate hypometabolism. Frontal hypometabolism was consistent with a diagnosis of AD if it was accompanied by more severe temporoparietal hypometabolism [27]. Patterns not compatible with AD (N-) included normal findings and patterns compatible with other neurodegenerative diseases. In cases of diagnostic discrepancies, a consensus diagnosis was reached.

### Clinical follow-up

Participants were followed up in our unit until January 2022. During follow-up visits, patients were subject to evaluation by a neurologist and a neuropsychological assessment. Progression to dementia due to AD was defined using the 2011 NIA-AA criteria [12].

### Statistical analyses

Descriptive statistical analyses were performed using SPSS (Statistical Package for Social Sciences, Version 23), while prospective and correlational statistical analyses were conducted using STATA (Software for Statistics And Data Science, Version 15). All analyses were adjusted for age, sex, and education level, with significance set at *P* < 0.05. Demographic data and other baseline characteristics were presented using descriptive statistical indices for the overall cohort and for each study group. Continuous variables were summarised using the mean and standard deviation (SD), while non-normal variables were described using the median and interquartile range (IQR), as appropriate. The differences in demographic characteristics between patients with aMCI who progressed and did not progress to AD were assessed using ANOVA or the Kruskal–Wallis test, as appropriate. The sensitivity, specificity, positive predictive value and negative predictive value were obtained for each test over 4 years. Kaplan–Meier survival curves were generated to estimate the probability of conversion over time based on the biomarker results.

## Results

### Population

This study recruited 145 patients with aMCI between October 2013 and March 2021; their demographic details and risk factors are described in Table 1. The mean age was 71.3 years (SD 6.2), and 59% were male. The average MMSE test score was 26.1 ± 6.2 points, while the average education level was 11.2 ± 4 years. Overall, 67.6% of patients were amyloid-positive, while 44.3% showed an AD pattern on FDG-PET.

**Table 1:** Demographic and clinical characteristics of the study population, converters and no converters to Alzheimer´s disease

	*Total aMCI (n = 145)*	*Converters (n = 75)*	*No Converters (n = 41)*	*p*-value
Age, Mean (SD)	71.32 (6.23)	71.34 (6.13)	71.26 (6.46)	0.940
Sex, Male %	59%	54%	67%	0.131
*MMSE score*,* Mean (SD)*	*26.17 (2.37)*	*25.84 (2.29)*	*26.74 (2.41)*	***0.025***
*Education*,* y*	*11.18 (3.98)*	*10.60 (4.01)*	*12.15 (3.75)*	***0.023***
*GDS score*	*7.14 (5.10)*	*7.34 (4.62)*	*8.80 (5.88)*	*0.554*
*Amyloid +*,* %*	*67.59%*	*95.59%*	*42.85%*	***< 0.0001***
*FDG +*,* %*	*44.36%*	*63.49%*	*27.14%*	***< 0.0001***
*Hypertension (yes) %*	*46.98%*	*45.58%*	*48%*	*0.767*
*Diabetes Mellitus (yes) %*	*15.17%*	*10.29%*	*19.48%*	*0.124*
*Dyslipidemia (yes) %*	*60.68%*	*61.76%*	*59.74%*	*0.803*
*Smoker (yes) %*	*8.96%*	*11.76%*	*6.49%*	*0.268*
*Ex-smoker (yes) %*	*33.10%*	*32.35%*	*33.76%*	*0.857*
*Antihypertensive use (yes) %*	*44.13%*	*41.17%*	*46.75%*	*0.500*
*Benzodiazepine (yes) %*	*31.03%*	*30.88%*	*31.16%*	*0.970*
*Antidepressives (yes) %*	*34.38%*	*35.29%*	*33.76%*	*0.847*
*Father AD (yes) %*	*2.75%*	*4.41%*	*1.29%*	*0.253*
*Mother AD (yes) %*	*9.65%*	*10.29%*	*9.09%*	*0.807*

Data are expressed as mean (SD) for continuous variables and as percentages for categorical variables. Statistical comparisons between AD converters and non-converters were performed with a p-value <0.05 considered statistically significant. Abbreviations: aMCI, amnestic mild cognitive impairment; AD, Alzheimer’s disease; MMSE, Mini-Mental State Examination; GDS, Geriatric Depression Scale; HTA, hypertension; DM, diabetes mellitus; SD, Standard Deviation; M, Male. Note: The totals for AD converters (*n* = 75) and non-converters (*n* = 41) represent patients with complete follow-up data (*n* = 116); 29 patients were lost to follow-up

### Conversion to AD dementia

**Fig. 1:**
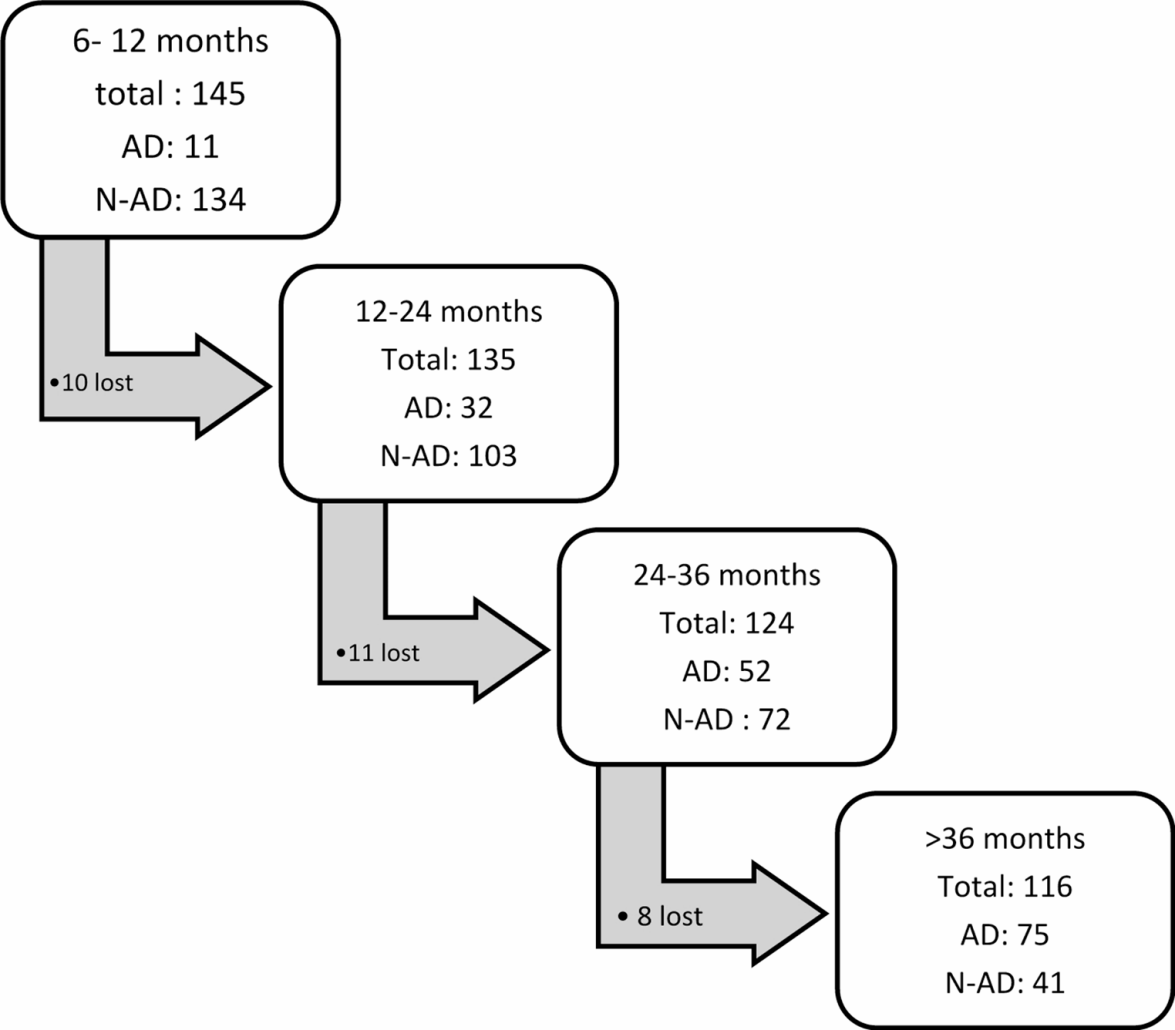
Flow diagram showing the longitudinal follow-up of 145 patients with amnestic mild cognitive impairment (aMCI) across successive intervals (6–12, 12–24, 24–36, and > 36 months). The number of individuals who progressed to Alzheimer’s disease (AD), remained clinically stable, or were classified as lost to follow-up is indicated at each time point. The category “lost” includes individuals who either discontinued follow-up visits or converted to a non-AD dementia during the observation period

On follow-up, 75 out of 116 patients progressed to dementia due to AD, while 29 patients were lost (i.e. lost to follow-up or converted to a different type of dementia). Details regarding follow-up and conversion rates are described in Fig. 1. The mean follow-up duration was 27.19 months (SD 18.334), ranging from 6 to 89 months. During the first 12 months, 11 patients progressed, 134 did not progress and 10 were lost. From 12 to 24 months, 32 patients progressed, 103 did not progress and 11 were lost. Between 24 and 36 months, 52 patients progressed, 72 did not progress and 8 were lost. After 36 months, 75 patients progressed, 41 did not progress and 8 were lost. Regarding demographics and risk factors, patients who progressed versus those that did not progress to dementia performed worse on the MMSE test (25.8 ± 2.2 vs. 26.7 ± 2.4; *p* = 0.025) and had fewer years of education (10.6 ± 4.0 vs. 12.1 ± 3.7 years; *p* = 0.023). Regarding PET scan results, a significantly greater percentage of patients who converted to AD had a positive Amyloid-PET (95.6%, *p* < 0.0001) and a positive FDG-PET (63.5%, *p* < 0.0001). Regarding the risk factors for dementia (i.e. diabetes mellitus, hypertension, dyslipidaemia, smoking, ex-smoking, antidepressant use, benzodiazepine use, antihypertensive drug use and family history of dementia), these had no significant differences when comparing based on biomarker results (all *p* > 0.05). (Table 2)

**Table 2:** Baseline demographic and clinical characteristics of the patients with available FDG PET data, stratified by amyloid and FDG PET status (AN, AN+, A + N, A + N+). Chi-square analyses of categorical variables revealed no statistically significant differences among the groups *p* > 0,05. Abbreviations: FDG, [18 F]-fluorodeoxyglucose; MMSE, Mini-Mental state examination; AD, Alzheimer’s disease

	AN (*n* 40)	AN+ (*n* 4)	A + *N* (*n* 34)	A + *N*+ (*n* 54)
Age in years, mean ± SD	71 ± 6.29	76 ± 5.39	73 ± 5.91	70 ± 6.40
Sex, Male (%)	(67.5)	(75)	(61.76)	(50)
Education Level				
No Formal Education, (%)	0 (0)	0 (0)	2.94 (1)	7.40 (4)
Primary education, (%)	16 (40)	2 (50)	14 (41.17)	17 (31.41)
Secondary education, (%)	7 (17.5)	2 (50)	10 (41.12)	15 (27.72)
Tertiary education, (%)	15 (37.5)	0	9 (26.47)	19 (35.18)
MMSE, (mean ± SD)	27 ± 2.16	26 ± 2.86	26 ± 2.09	26 ± 2.24
Hypertension (%)	19 (47.5)	1 (25%)	20 (58.82)	22 (40.74)
Diabetes Mellitus %	9 (22.5)	0 (0)	4 (11.76)	5 (9.25)
Dyslipidemia, (%)	25 (62.5)	3 (75)	23 (67.64)	30 (55.55)
Current Smoker, (%)	3 (7.5)	0 (0)	4 (11.76)	5 (9.25)
Ex-Smoker, (%)	14 (35)	1 (25)	8 (23.52)	20 (37.03)
Antihypertensive Therapy, (%)	18 (45)	2 (50)	18 (52.94)	21 (38.88)
Benzodiazepine Use, (%)	15 (37.5)	1 (25)	10 (29.41)	16 (29.62)
Antidepressant Use, (%)	19 (47.5)	2 (50)	8 (23.53)	19 (35.19)
Family History of AD (Father), (%)	1 (2.5)	0 (0)	0 (0)	2 (3.70)
Family History of AD (Mother), (%)	1 (2.5)	0 (0)	5 (14.71)	6 (11.11)

### Accuracy of amyloid-PET and FDG-PET to estimate the conversion to dementia due to AD

Table 3 shows the sensitivity, specificity, positive predictive value and negative predictive value of Amyloid-PET and FDG-PET over the years.

**Table 3:** Evolution of diagnostic performance parameters for Amyloid-PET and FDG-PET in predicting conversion to Alzheimer’s disease. Data are presented as sensitivity, specificity, PPV (positive predictive value), and NPV (negative predictive value at 12, 24, 36, and 48 months of follow-up

	*Amyloid PET*				*FDG PET*			
	Sensitivity (%)	Specificity (%)	PPV (%)	NPV (%)	Sensitivity (%)	Specificity (%)	PPV (%)	NPV (%)
*12 months*	100	35	11.2	100	60	57	10	94.6
*24 months*	100	43.7	35.5	100	70	64.4	38	87.3
*36 months*	98.1	52.7	60	97.4	68	70.6	61.5	76.2
*48 months*	94.6	73	86.6	88	62.8	85	88	56.6

### Amyloid-PET

At years 1 and 4 of follow-up, respectively, Amyloid-PET demonstrated a sensitivity of 100% and 94.67%, as well as a negative predictive value of 100% and 88.24% for predicting conversion to AD dementia. The specificity and positive predictive value tended to increase over time. From 12 to 48 months, specificity increased from 35.07 to 73.17%, while positive predictive value increased from 11.22 to 86.59%.

### FDG-PET

At years 1 and 4 of follow-up, respectively, FDG-PET exhibited a negative predictive value of 94.59% and 87.32%. Throughout the follow-up period, both specificity and positive predictive value increased (85% and 88%, respectively, after 48 months). Meanwhile, sensitivity was generally constant, starting at 60% in the first 12 months then remaining at 62.86% after 48 months.

### Combinations of amyloid-PET and FDG-PET

**Fig. 2:**
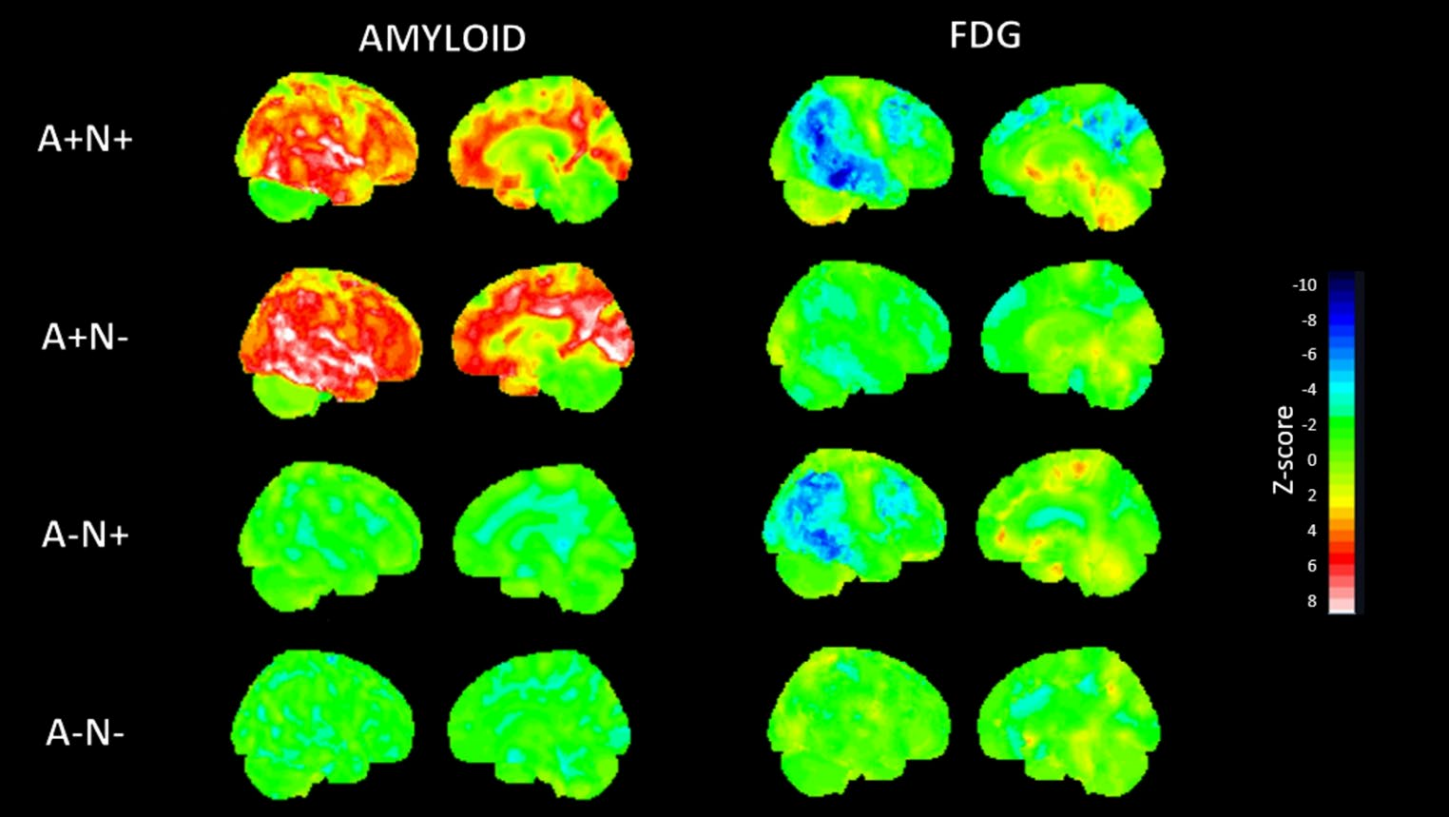
Representative Z-score maps derived from amyloid-PET and FDG-PET imaging across four biomarker-defined AT(N) groups: A + N+, A + N–, A–N+, and A–N–. Sagittal brain views show cortical amyloid burden (left column) and glucose hypometabolism (right column). Z-scores were computed using a normative database of cognitively unimpaired individuals. Increased amyloid uptake (Z > 2) is shown in red-white hues, while reduced FDG uptake (Z < − 2) is represented in blue. All images were spatially normalized to MNI space and visualized using a standard intensity scale

Patients were divided into four groups based on the results of Amyloid-PET (amyloid-positive [A+] or amyloid-negative [A-]) and FDG-PET (suggestive of AD [N+] or not [N-]): A+/N + group (*n* = 54), A+/N- group (*n* = 34), A-/N + group (*n* = 4) and A-/N- group (*n* = 40)(Fig. 2)). Due to the small size of the A-/N + group, it was merged with the A-/N- group for some analyses.

**Fig. 3:**
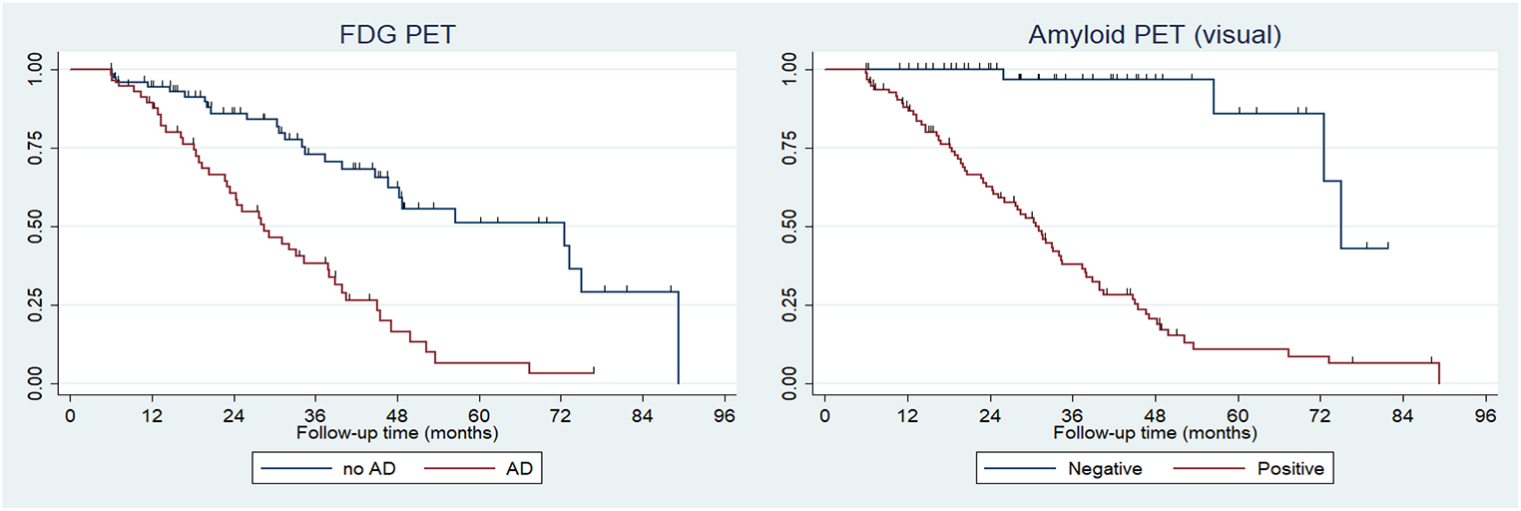
Kaplan–Meier survival curves illustrating the risk of clinical progression to AD based on FDG-PET (left) and visual amyloid-PET (right) status. Time to progression is expressed in months from baseline assessment

The median rate of conversion to AD was 39.95 months, which differed based on biomarker results. Amyloid-negative patients (*n* = 47) exhibited delayed progression, with a mean of 75.07 months (95% CI: 56.54–81.00), among which 3 patients (6.4%) progressed to AD by the end of the follow-up period. Meanwhile, amyloid-positive patients exhibited a significantly more rapid progression, with a mean of 32.59 months (95% CI: 20.56–40.74). Approximately 25% of amyloid-positive patients converted within the first 24 months, and this rate steadily increased, reaching nearly 100% by the end of follow-up (96 months), representing a markedly steeper decline compared to the amyloid-negative group (hazard ratio [HR]: 5.007 [95% CI: 2.521–9.942]; *p* < 0.0001) (Fig. 3).

Patients with a negative result on FDG-PET exhibited a later progression to AD, with a mean of 31.96 months (SD = 21.175). More than 75% did not progress to AD for the first 36 months, and although this proportion gradually declined over time, it remained above 50% at the end of the follow-up period. Meanwhile, patients with an FDG-PET pattern consistent with AD exhibited earlier progression, with a mean of 25.03 months (SD = 15.400). Approximately 50% of patients progressed before 36 months, and almost 100% progressed by 72 months representing a significantly steeper decline versus FDG-negative patients (HR: 3.127 [95% CI: 1.82–5.36]; *p* < 0.001) (Fig. 3).

**Fig. 4:**
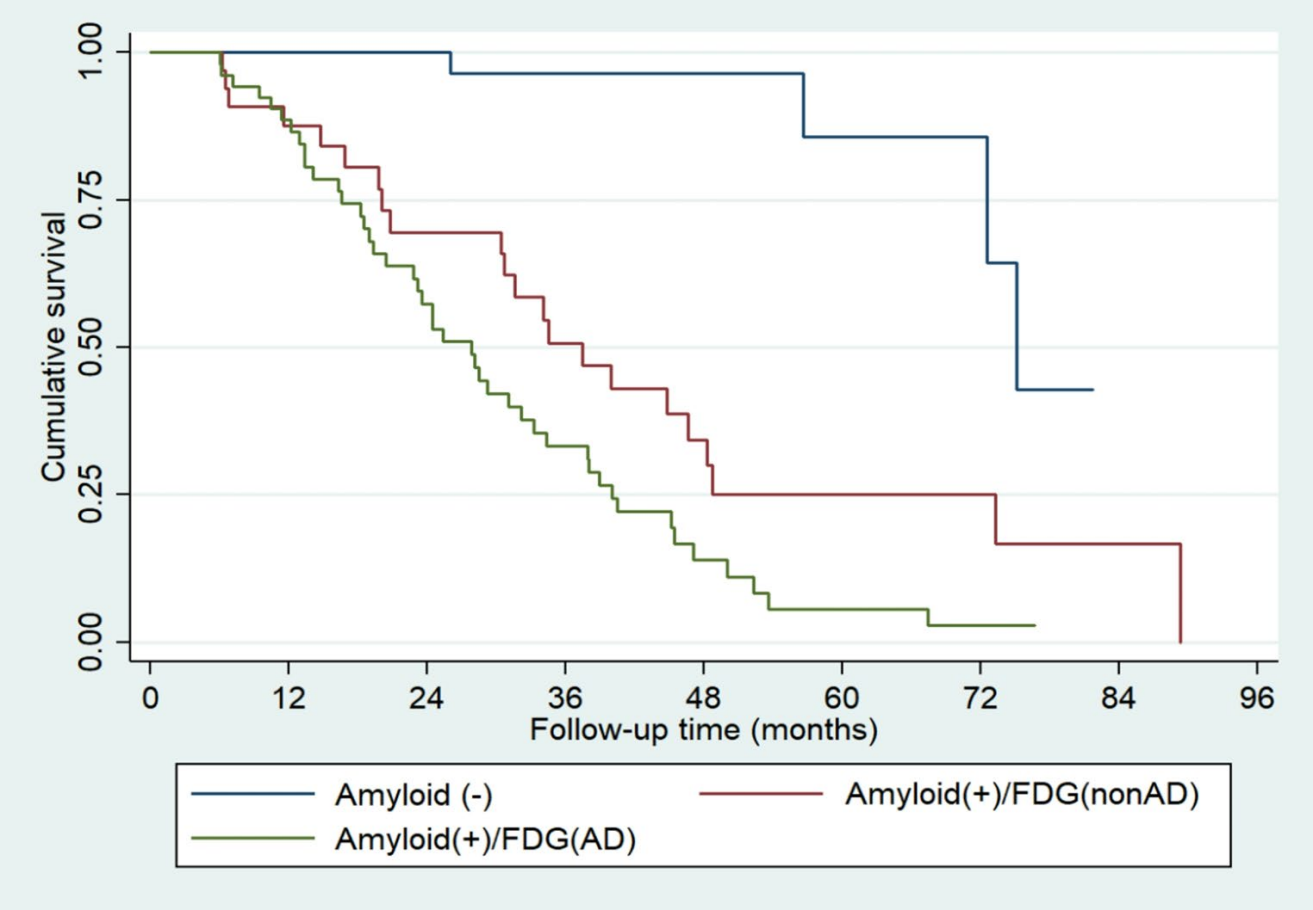
Kaplan–Meier survival curves illustrating time to clinical progression to AD according to combined amyloid and FDG-PET biomarker profiles. The blue curve represents individuals with a negative amyloid-PET scan (A–), the red curve represents amyloid-positive individuals without AD-consistent FDG hypometabolism (A + N–), and the green curve corresponds to subjects with both amyloid and FDG-PET positivity (A + N+). Time is expressed in months from baseline, with risk of progression increasing across the three groups

Combining both PET scan findings, A+/N + patients converted significantly earlier than A+/N- patients (median: 27.80 [CI 2.70–20.40] vs. 37.38 (CI 20.73–48.26) months; chi2 = 4.16, *p* = 0.0415) (Fig. 4).

## Discussion

Several studies have demonstrated the utility of PET imaging biomarkers in predicting the early progression from aMCI to AD dementia, which is important in the context of initiating disease-modifying treatment [28–32]. This longitudinal study of nearly 8 years demonstrates the utility of Amyloid-PET and FDG-PET biomarkers in clinical practice among patients with aMCI for the early prediction of progression to AD dementia. Both Amyloid-PET and FDG-PET played significant roles in predicting conversion to AD among patients with aMCI. In line with this, a recently published systematic review affirmed that FDG-PET and Amyloid-PET were the most accurate molecular imaging biomarkers in predicting conversion from MCI to AD [33]. Additionally, our results demonstrated that the use of both biomarkers at the start of follow-up strongly predicted short-term conversion to AD, which can be highly valuable for clinicians.

Amyloid-PET and FDG-PET each play different roles in predicting conversion to AD. Amyloid-PET exhibited very high sensitivity (100% in year 1, 94.67% in year 4) and negative predictive value (100% in year 1, 88.24% in year 4), which is consistent with previous findings [31]. A positive Amyloid-PET result in the early stages of the disease is known to predict conversion to AD dementia. Our study confirms these findings, as 95.6% of patients who converted to AD within 8 years were amyloid-positive (*p* > 0.0001), and these patients converted 42.48 months earlier than amyloid-negative patients (*p* < 0.0001). Similarly, previous research has demonstrated that Amyloid-PET exhibits high sensitivity (95% within a 4-year period) but low specificity (33% at baseline, increasing to 73% on follow-up) for predicting progression [31]. However, FDG-PET had a very high negative predictive value from the beginning of follow-up in our study (94.59% in year 1, 87.32% in year 4), meaning that patients with a negative finding on FDG-PET did not exhibit early conversion. However, the specificity of FDG-PET increased over time, with conversion seen after 48 months in 85% of patients with a positive FDG-PET.Similarly, Sanchez-Catasus et al. [34] found that FDG-PET appeared to predict the early conversion of aMCI patients. Moreover, Jagust et al. concluded that FDG-PET results can alter the probability of a clinical diagnosis matching the postmortem final diagnosis of AD. They found that the initial positive clinical diagnosis for AD had an accuracy of 70% compared to the anatomopathological diagnosis, but this increased to 84% with a positive FDG-PET, while a negative FDG-PET reduced the probability to 31%. Conversely, the probability of AD with a negative clinical diagnosis was 35%, but this was decreased to 17% with a negative FDG-PET and increased to 70% with a positive FDG-PET [28].

The duration of follow-up plays an important role in predicting conversion to dementia due to AD. Previous studies have significant variations in the duration of follow-up, ranging from approximately 1 year to more than 5 years. The prevailing consensus is that prolonged follow-up of patients with aMCI is associated with an increased likelihood of meeting the criteria for dementia. However, the sensitivity of biomarkers does not depend on the follow-up time, as reported by Barthel [29]. Our data agrees with these previous findings, since the very high sensitivity of Amyloid-PET (95–100%) and moderate sensitivity of FDG-PET (62.8–70%) both remained stable over the years.

Our results confirm that, among aMCI patients, a positive Amyloid-PET result predicts conversion to dementia due to AD at some point during the disease, while a negative FDG-PET result supports non-conversion, consistent with previous studies. Notably, a small proportion of amyloid-negative patients progressed in the later stages of follow-up, which is consistent with previous reports indicating that up to 15% of patients with clinically diagnosed AD may have negative Amyloid-PET scans ( [35, 36].). Several hypotheses have been proposed to explain this phenomenon. One possibility is that these patients could have an atypical or non-amyloid AD pathology, where neurodegeneration occurs despite the absence of significant amyloid deposition [37]. Another possibility involves limbic-predominant amnestic neurodegenerative syndrome (LPA-ND) [38], a distinct clinicopathological entity that is characterised by hippocampal atrophy and tau pathology without substantial cortical amyloid accumulation. This could explain why some patients meeting clinical the criteria of AD do not exhibit amyloid deposition on PET imaging. These cases highlight the complexity of the pathological spectrum underlying neurodegenerative diseases. These cases would greatly benefit from a multimodal diagnostic approach that incorporates FDG-PET, hippocampal atrophy assessment, and CSF biomarkers to improve diagnostic accuracy. Further research is needed to elucidate the underlying mechanisms of amyloid-negative phenotypes of AD, as well as to determine whether LPA-ND is a different disease or a subset of AD with distinct pathophysiological characteristics. Understanding these cases is crucial for refining diagnostic criteria and guiding treatment strategies for patients with atypical presentations of neurodegeneration.

One of our main objectives was to determine the implications of using both Amyloid-PET and FDG-PET in combination, as well as whether ordering both at the start of follow-up could influence our clinical approach. Based on our findings, ordering both tests early can help prognosticate the patient in terms of their likelihood of early conversion to AD. While having a positive Amyloid-PET indicates a high probability of progression to AD, additionally having a positive FDG-PET result further indicates progression at an earlier onset and to a greater degree. When considering the FDG-PET results from the beginning, we observed that a positive FDG-PET predicts early progression. In our cohort, A+/N + patients progressed earlier and to a greater extent versus A+/N- patients (mean: 27.79 [CI 20.40–33.21] vs. 37.38 [CI 20.73–48.26] months). In the published guidelines for using biomarkers among patients with cognitive impairment, three types of diagnostic pathways were highlighted. In our case, pertaining to patients with aMCI and suspected AD, the guidelines recommend an additional FDG-PET study in cases wherein Amyloid-PET is positive and there is a desire to confirm the possibility of early conversion [6]. Our findings support this recommendation of using FDG-PET after a positive Amyloid-PET or even simultaneously to predict early progression in patients with aMCI, especially because of the earlier progression if both are positive.

The limitations of this study must be discussed. The data in this study was drawn from a clinical setting, which can differ from those acquired from a research setting. Additionally, there was no control group, and the number of patients in each group was unequal. Furthermore, the clinical follow-up period in the present study varied between patients, which could be considered a limitation especially among those that did not exhibit progression. Lastly, the aetiological diagnosis was determined based on the patients’ clinical progression during the follow-up period without a postmortem confirmation of the diagnosis.

## Conclusion

Both Amyloid-PET and FDG-PET are valuable tools for establishing prognosis in patients with aMCI in clinical practice. Having a positive Amyloid-PET was associated with an increased risk of progression to dementia due to AD. Furthermore, amyloid-positive patients who also have a pattern of AD on the FDG-PET study exhibited earlier progression to AD dementia versus those with a positive Amyloid-PET alone. Thus, the use of both biomarkers during the initial diagnostic process can aid clinicians in predicting short-term progression to AD.

## Data Availability

The datasets generated during and/or analysed during the current study are available from the corresponding author on reasonable request.
